# Prevalence of common mental disorders and associated factors among adults living in Harari regional state, eastern Ethiopia: a community based cross-sectional study

**DOI:** 10.3389/fpsyt.2023.1183797

**Published:** 2023-07-13

**Authors:** Tilahun Abdeta, Abdi Birhanu, Haregeweyn Kibret, Ayichew Alemu, Kefelegn Bayu, Kasahun Bogale, Fentahun Meseret, Deribe Bekele Dechasa, Fenta Wondimneh, Yideg Abinew, Magarsa Lami, Dawud Wedaje, Tilahun Bete, Kabtamu Gemechu, Shambel Nigussie, Abraham Negash, Gebisa Dirirsa, Bekelu Berhanu, Jemal Husen, Addis Eyeberu, Abduro Godana, Yadeta Dessie, Addisu Sertsu, Ahmed Hiko, Henock Asfaw, Jerman Dereje, Kabtamu Nigussie

**Affiliations:** College of Health and Medical Sciences, Haramaya University, Harar, Ethiopia

**Keywords:** common mental disorders, prevalence, associated factors, community, Ethiopia

## Abstract

**Background:**

Common mental disorders are highly prevalent in the population, affecting people across all regions of the world. In Ethiopia, mental disorders are the leading non-communicable disorders. World Health Organization (WHO) report shows that 4,480,113 (4.7%) and 3,139,003 (3.3%) people in Ethiopia are estimated to suffer from depression and anxiety, respectively. However, there are only limited studies conducted on common mental disorders in Africa including Ethiopia. Even if there was a previous study conducted among Harari regional state residents 7 years back, nowadays there are many possible factors that could increase the prevalence of common mental disorders in the community like the novel coronavirus pandemic, ethnic war, and current socio-economic crises in Ethiopia.

**Objective:**

To assess the prevalence of common mental disorders and associated factors among adult residents of Harari regional state, eastern Ethiopia.

**Methods:**

A community-based cross-sectional study was conducted from March 1 to March 30, 2022. A systematic random sampling technique was used to select 1,192 study participants. Data were collected by interviewer-administered structured and semi-structured questionnaires. A common mental disorder was assessed by the Self-Reporting Questionnaire of 20-Item (SRQ-20) questionnaire. Data were entered into Epi Data version 3.2 and exported to Stata Version 16 for analysis. The multivariable binary logistic regression analysis with a 95% confidence interval and *p* < 0.05 was used to identify factors associated with common mental disorders.

**Result:**

From a total of 1,192 eligible participants, 1,168 responded to this survey, giving a response rate of 97.98%. The prevalence of common mental disorders among adults in the Harari Region of this study was 21.31% (95% CI, 18.8–23.60). Being female with Adjusted Odds Ratios (AOR = 1.31, 95%CI, 1.09–2.09), single (AOR = 2.03, 95%CI, 1.37–3.01), divorced (AOR = 3.06, 95%CI, 1.97–4.76), widowed (AOR = 1.79, 95%CI, 1.23–3.26), unemployed (AOR = 1.97, 95%CI, 1.18–3.31), having family history of mental illness (AOR = 3.17, 95%CI, 1.54–6.96) and age greater or equal to 55 years (AOR = 105, 95%CI, 1.69–3.54) were statistically associated with common mental disorders.

**Conclusion:**

The prevalence of common mental disorder in the study area was sharply increased. Being female, single, divorced, widowed, family history of mental illness, being unemployed and age greater or equal to 55 years old were statistically positively associated with common mental disorder. Considering these determinants, screening, early identification, and providing appropriate intervention for common mental disorders in the community should be of great concern.

## Introduction

Mental health is a state of well-being in which every individual realizes his or her potential, can cope with the normal stresses of life, can work productively and fruitfully, and can contribute to her or his community (1). Common mental disorders (CMD) indicate the more prevalent mental health problems and are characterized by a range of anxiety and depressive disorders which have a long-term impact on human life (2).

Common mental disorders are highly prevalent in the population, affecting people across all regions of the world (3). The global burden of mental illness accounts for 32.4% of years lived with disability (YLDs) and 13.0% of disability-adjusted life-years (DALYs) (4). Of the global burden, almost three-quarters of the burden lies in low-and middle-income countries (LMICS) (5). Globally, 300 million (4.4%) and 264 million (3.6%) people are estimated to suffer from depression and anxiety, respectively, and more than 80% of this burden occurred in low-and middle-income countries (2).

There are limited studies conducted on common mental disorders in Africa. Besides, the available studies reported different figures from different countries. A population-based epidemiological survey of a rural area in Kenya revealed the point prevalence of CMD was 10.8%, largely comprising mixed anxiety-depression (6.1%), panic disorder (2.6%), generalized anxiety disorder (1.6%), and depressive episodes (0.7%) (6). The other population-based studies in Ghana and South Africa showed that the prevalence of CMD among adult residents was 51.8% and 25.0%, respectively (7, 8).

In Ethiopia, mental disorders are the leading non-communicable disorders (9). World Health Organization (WHO) report shows that 4,480,113 (4.7%) and 3,139,003 (3.3%) people in Ethiopia are estimated to suffer from depression and anxiety respectively; the total years lived with a disability was about 837,683 (10.1%) led by depressive disorder and 292,650 (3.6%) by anxiety disorder (2). Previous cross-sectional community-based studies in Jimma Town, South West Ethiopia, Illu Ababor zone, southwest Ethiopia, Silte Zone, Southern Ethiopia, in Addis Ababa, and Harari Regional State, Eastern Ethiopia reported that the prevalence of CMD among adult residents was 33.6%, 27.2%, 39.7%, 24.7%, and 14.9%, respectively (10–14). These past studies more focused on the southern part of Ethiopia and could not give national representative data. Ethiopia has many regional states with different socio-cultural contexts. Therefore, different studies across the country are expected to provide nationally representative evidence. Besides, in Harari regional state the studies are very limited. The only available population-based study on CMD in this region was conducted around 6 years back (14) and studies with current evidence are needed.

Individuals with an untreated CMD have increased odds of medical illness, suicide, and early mortality (15). Individuals with CMD cannot perform their occupation adequately and they are highly experiencing low workability (16). It has an impact on the mood or feelings of affected persons; the severity of the symptoms ranges from mild to severe and it stays from months to years. It causes marked emotional distress and interferes with daily function (17).

On the other hand, different variables were reported as having a significant association with common mental disorders in the community. Some of them include lower socioeconomic status, history of psychological illnesses, poor reproductive health, gender disadvantage, physical ill-health, Smoking and sedentary behavior, older age, being female, housewife, educational status (unable to read and write), and chewing Khat (10, 11, 18).

Generally, even if there was a previous study conducted among Harari regional state residents 6 years back (14), nowadays there are many possible factors that could increase the prevalence of common mental disorders in the community like the novel coronavirus pandemic, ethnic war, and current socio-economic crises in Ethiopia; even though these variables were not incorporated into the independent factors of this study. So, it is important to know the current prevalence using a representative sample of the population. The public health significance of mental and behavioral disorders is demonstrated by the fact that they are among the most important causes of morbidity in primary care settings and produce considerable disability. Therefore, this study aimed to assess the prevalence of CMD and associated factors among adult residents in Harar Town of Harari regional state, eastern Ethiopia.

## Materials and methods

### Study setting, design, and period

A community-based quantitative cross-sectional study was conducted from March 1 to March 30, 2022, in Harari regional state, which is located 510 km away from the capital, Addis Ababa. The region is divided into nine districts, with three of them being rural and six being urban. The urban districts are subdivided into 19 kebeles (the lowest administrative division in the country), and the rural districts are subdivided into 17 peasant associations (which is equivalent to kebeles in the urban case).

### Population and eligibility criteria

Harari regional state residents were a source population. All residents living in randomly selected kebeles of Harari regional state with age greater or equal to 18 years were a study population. Residents living in randomly selected households as heads or any other household members greater than or equal to 18 years of age and residents living more than 6 months and available during data collection were included in the study. Peoples who left the house for some reason and who were seriously ill during the data collection period were excluded.

### Sample size determination and sampling procedure

The sample size was calculated by using a single population proportion formula with the following statistical assumptions: *n* = the minimum sample size required, *p* = the estimated proportion of CMD, *z* = the standard value of confidence level of alpha = 95%, *d* = the margin of error between the sample and the population (0.03). For this study *p* = 14.9% (the prevalence of common mental disorders from a similar study) was used (14).


n=(Zα2)2P(1−P)d2

$$n=\frac{{\left(1.96\right)}^{2}\;0.149\;\left(1-0.149\right)}{0.03^{2}}=542$$

Accordingly, with a design effect of two and adding a 10% non-response rate, the final sample size was 1,192. A multi-stage random sampling technique was used in sampling the study participants. From 9 districts, 13 kebeles were selected using a simple random sampling (lottery) method. Then, from selected kebeles, 1,192 households were allocated proportionally. Each study unit (HH) was selected using a systematic random sampling method. Then, the eligible individual within the selected household was selected using a simple random sampling method.

### Data collection procedure and tools

Face-to-face interviews were used to collect data using a semi-structured questionnaire. The questionnaire contains four parts, which are socio-economic characteristics of the patients adapted and modified from reviewing similar literature, and clinical, psychosocial, and substance-related factors. Common mental disorders were assessed by using the Self-Reporting Questionnaire of 20-Item (SRQ-20) developed by the World Health Organization. It has 20 questions that are answered by YES or NO with codes “1” which represent the presence of a symptom, and “0” if the symptom is absent. If the submission of the respondent answers more than six of the twenty questions the respondent was considered to have common mental disorders (19–21). With a cut-off score of 6, the positive predictive value of SRQ-20 is 83.9%. This assessment instrument (SRQ 20) was validated in Ethiopia. It showed better validity as a dimensional scale, with area under the receiver operating characteristic (AUROC) of 0.82 (95%CI, 0.68–0.96) and 0.70 (95%CI, 0.57–0.83) in two different validation studies. The internal consistency, measured by Cronbach’s alpha, was excellent in both validation studies: 0.84 (study 1), 0.88 (study 2) (22). The SRQ-20 in this study showed a high internal consistency reliability coefficient (Cronbach’s alpha = 0.86).

Social support was assessed by the Oslo social support scale (Oslo-3) which contains three items. It is a 3 item questionnaire, commonly used to assess social support and it has been used in several studies. The sum score scale ranged from 3 to 14, which had three categories: poor support 3–8, moderate support 9–11, and strong support 12–14 (23), and it was validated in Ethiopia (24).

Substance-related factors were assessed by Alcohol, Smoking, and Substance Involvement Screening Test (ASSIST), which is a brief screening questionnaire developed and validated by the World Health Organization (WHO) to find out about people’s use of psychoactive substances. It was used to assess the current and ever substance use history of the participants (25). Data were collected by 10 BSc psychiatric nurses and supervised by 2 mental health professionals who were master holders in integrated clinical and community mental health.

## Study variables

### Dependent variable

Status of common mental disorders (yes/no).

### Independent variables

**Socio-demographic variables** (sex, age in years, marital status, religion, occupational status, educational status, residence, and monthly income in Ethiopian birr), **clinical factors** (family history of mental illness, emotional stress, and comorbid medical illness), **substance-related factors** (current and lifetime substance use of alcohol, tobacco, khat, and cannabis/ hashish), and **psychosocial factor** (perceived social support).

### Data quality control

Data collectors and supervisors were trained for 1 day on the data collection approach of the study. The questionnaire was translated into local languages, Amharic and Afan Oromo, by an expert and back-translated into English by another person to check for consistency. A pretest was conducted among 60 adults (5% of the sample size) living in Haramaya town, which is nearest to the study areas but not part of the study areas, to see the applicability of the instruments, and feedback was incorporated into the final tool to improve the quality. Supervision was done by the supervisors and principal investigator throughout the data collection period and checked daily for completeness and consistency of questioners.

### Data processing and analysis

The data were coded, cleaned, and entered into Epi Data version 3.2 and then exported to Stata version 16 for analysis. Bivariable and multivariable logistic regression analysis was performed to identify factors associated with a common mental disorder. All variables with a *p*-value less than 0.20 in bivariate analysis were entered into the multivariable logistic regression analysis. To estimate the strength of association between an outcome and explanatory variables, an adjusted odds ratio (AOR) with a 95% confidence interval was reported. A *p*-value of less than 0.05 is considered statistically significant. Multi-collinearity was assessed by the variance inflation factor (VIF) to see a correlation between independent variables and VIF were less than 5 for all variables, indicating that there was no problem of multicollinearity between the predictor variables. The goodness of model fitness was checked by using the Hosmer-Lemeshow test.

### Ethical considerations

Ethical clearance was obtained from the Institutional Health Research Ethics Review Committee (reference number: IHRERC/043/2022) of the College of Health and Medical Sciences of Haramaya University. Then data collection was initiated after a letter of permission was obtained from Haramaya University College of Health and Medical Sciences for each district and Kebeles administrator. Official permission was secured from woreda and the Keble administrator. Also, informed, voluntary, written and signed consent was obtained from each participant after explaining the purpose and importance of the study before the interviews. Participants were informed about the aim of the study and the advantage of the study; confidentiality, as there was no risk of being participants, and they have full right to halt in the middle of the interview. Written informed consent was taken from each participant before data collection began. Confidentiality was maintained at all levels of the study through anonymous data collection. During data collection, the COVID-19 prevention protocol was kept.

## Results

### Socio-demographic characteristics of participants

A total of 1,168 participants were included in the study with a response rate of 97.98%. The median age of respondents was 40, with an interquartile range (IQR, 30–65) years. More than half of the participants, 57.87% (676) were males and around two-third, 64.47% (753) were married. Regarding the educational status of participants 30.65% (358) were able to read and write, the majority of participants 46.49% (543) were Muslim religion followers and nearly three-fourth, 72.43% (846) were urban residents as show in Table 1. Generally, the socio-demographic variations found in Table 1 is the matter of chance and does not reflect the actual population of the study area/region.

**Table 1:** Socio-demographic and economic distributions of study participants in Harari regional state, Eastern Ethiopia (*n* = 1,168).

Variables	Categories	Frequency (*n* = 1,168)	Percentage (%)
Sex	Male	676	57.88
	Female	492	42.12
Age (years)	18–24	131	11.21
	25–34	171	14.64
	35–44	190	16.26
	45–54	153	13.12
	≥55	523	44.77
Marital status	Married	753	64.45
	Single	187	16.01
	Divorced	118	10.1
	Widowed	110	9.42
Religion	Muslim	543	46.49
	Orthodox	401	34.33
	Protestant	121	10.36
	Others^a^	103	8.82
Occupational status	Government worker	319	27.31
	Merchant	269	23.03
	Student	153	13.1
	Farmer	161	13.78
	Daily labor	48	4.11
	Household worker	103	8.82
	Unemployed	115	9.85
Educational status	Unable to read and write	183	15.67
	Able to read and write	358	30.65
	Primary (1–8)	203	17.38
	Secondary (9–12)	221	18.92
	Diploma and above	203	17.38
Residence	Rural	322	27.56
	Urban	846	72.44
Monthly income in Ethiopian Birr	≤1,499	263	22.51
	1,500–2,999	265	22.69
	3,000–4,999	285	24.4
	≥5,000	355	30

a Catholic, Wakefata, and Adventist.

### Clinical, psychosocial, and substance-related characteristics of respondents

Most of the participants, 97.17% (1135) reported no family history of mental illness. Around half 46.15% (539) of respondents were lifetime substance users and 34.59% (404) were chewing Khat during the last 3 months. But only 1.3% (15) uses hashish/cannabis during the last 3 months. Out of all study respondents, around 27.65% (323) and 33.73% (394) had a comorbid medical illness and moderate social support, respectively, as shown in Table 2.

**Table 2:** Clinical, substance use, and psychosocial features of study participants in Harari Regional State, Eastern Ethiopia (*n* = 1,168).

Variables	Categories	Frequency (*n* = 1,168)	Percent (%)
Family history of mental illness	Yes	33	2.83
	No	1,135	97.17
Emotional stress	Yes	219	18.75
	No	949	81.25
Comorbid medical illness	Yes	323	27.65
	No	845	72.35
Lifetime substance use	Yes	539	46.14
	No	639	53.86
Current alcohol use in the last 3 months	Yes	123	10.53
	No	1,045	89.47
Current Khat use in last 3 months	Yes	404	34.58
	No	764	65.42
Current smoking cigarettes in the last 3 months	Yes	175	15
	No	993	85
Current use of cannabis/hashish in the last 3 months	Yes	15	1.28
	No	1,153	98.72
Social support	Poor	397	34
	Moderate	394	34.73
	Strong	377	32.27

### Prevalence of common mental disorders among adults living in Harari regional state

In this study, participants who scored more than 6 of the 20 questions of SRQ-20 were considered as having common mental disorders. Therefore, using this cut-off point (> 6 of SRQ-20), the prevalence of common mental disorders among adults living in the Harari regional state was 21.3% (95%CI, 18.8–23.6). The prevalence of common mental disorder was higher among female residents (24.79%) than male residents (18.78%), widowed (26.36%) than married (15.94%), among unemployed (38.26) than government employer (18.18), older age of ≥55 years (24.47%) than youngers of 15–24 years old (14.50%), current Alcohol users (29.27%) than non-users (20.38%), and among those who had family history of mental illness (45.45%) than who had not it (20.62%). The SRQ-20 responses (yes/No percentages) by the respondents was illustrated in Table 3.

**Table 3:** Self-reported questions responses of study participants living in Harari region state, Eastern Ethiopia (*n* = 1,168).

SRQ-20 questions	Yes/No	Frequency (*n* = 1,168)	Percentage (%)
1. Often have head ache	Yes	284	24.3
	No	884	75.7
2. Have poor appetite	Yes	174	14.9
	No	994	85.1
3. Sleep badly	Yes	199	17.0
	No	969	83.0
4. Easily frightened	Yes	128	11.0
	No	1,040	89.0
5. Have shaking hands	Yes	140	12.0
	No	1,028	88.0
6. Feel nervous, tense, or worried	Yes	164	14.0
	No	1,004	86.0
7. Have poor digestion	Yes	164	14.0
	No	1,004	86.0
8. Have trouble thinking clearly	Yes	145	12.4
	No	1,023	87.6
9. Feeling unhappy	Yes	161	13.8
	No	1,007	86.2
10. Cry more than usual	Yes	146	12.5
	No	1,022	87.5
11. Find difficult to enjoy your daily activities	Yes	150	12.8
	No	1,018	87.2
12. Find it difficult to make decisions	Yes	151	12.9
	No	1,017	87.1
13. Your daily work suffering	Yes	152	13.0
	No	1,016	87.0
14. Unable to play a useful part in life	Yes	151	12.9
	No	1,017	87.1
15. Lost interest in things	Yes	150	12.8
	No	1,018	87.2
16. Feeling as a worthless person	Yes	134	11.5
	No	1,034	88.5
17. Thought of ending your life been on your mind	Yes	133	11.4
	No	1,035	88.6
18. Feeling tired all the time	Yes	155	13.3
	No	1,013	86.7
19. Uncomfortable feelings in your stomach	Yes	104	8.9
	No	1,064	91.1
20. Easily tired	Yes	146	12.5
	No	1,022	87.5

### Factors associated with common mental disorders among adults living in the Harari region

In bivariate logistic regression analysis variables like being female, single, divorced, widowed family history of mental illness, current alcohol use in the last 3 months, students, unemployed, age greater or equal to 55 years were significantly associated with common mental disorders. However, in the multivariate logistic regression analysis variables like a female, single, divorced, widowed, family history of mental illness, unemployed, and age greater or equal to 55 years were statistically significantly associated with common mental disorders with a *p*-value less than 0.05.

In this study, the odds of having CMD among respondents with were female were about 1.31 times higher as compared to participants those being male [AOR = 1.31 (95%CI, 1.09–2.09)], and the odds of having common mental disorders among participants who were widowed, single, and divorced were 1.79, 2.03 and 3.06 times higher as compared to respondents who were married with [AOR = 1.79 (95%CI, 1.23–3.26)], [AOR = 2.03 (95%CI, 1.37–3.01), and AOR = 3.06 (95%CI, 1.97–4.76)] respectively.

The odds of having common mental disorders among respondents who had a family history of mental illness were 3.21 times higher as compared to respondents who had no family history of mental illness [AOR = 3.17 (95%CI, 1.54–6.96)] and odds of having common mental disorders among participants who were unemployed was 1.97 times higher as compared to participants who were government employers [AOR = 1.97 (95%CI, 1.18–3.31)]. Besides, the odds of having common mental disorders among participants who were age greater or equal to 55 years was 1.05 times higher as compared to respondents who were found within the age category of 15–24 [AOR = 1.10 (95%CI, 1.69–3.54)] as shown in Table 4.

**Table 4:** Factors associated with common mental disorders in multivariate and bivariate logistic regression analysis among study participants living in Harari region state, Eastern Ethiopia (*n* = 1,168).

Explanatory variables	Common mental disorders		COR (95%CI)	AOR (95%CI)
	Yes	No		
Sex				
Female	122	370	1.43 (1.08–1.89)	1.31 (1.09–2.09)*
Male	127	549	1	1
Marital status				
Married	120	633	1	1
Single	56	131	2.25 (1.56–3.26)	2.03 (1.37–3.01)**
Divorced	44	74	3.14 (2.06–4.78)	3.06 (1.97–4.76)**
Widowed	29	81	1.89 (1.18–3.01)	1.79 (1.23–3.26)*
Occupational status				
Government employer	58	261	1	1
Merchants	43	226	0.86 (0.56–1.32)	0.74 (0.45–1.20)
Students	41	121	1.65 (1.04–2.60)	1.48 (0.91–2.41)
Farmer	35	126	1.25 (0.78–2.06)	1.02 (0.61–1.71)
Daily labour	7	41	0.77 (0.33–1.80)	0.62(o.26–1.50)
House wife	21	82	1.15 (0.66–2.01)	0.91 (0.49–1.64)
Unemployed	44	71	2.79 (1.74–4.47)	1.97 (1.18–3.31)*
Age (in years)				
15–24	19	112	1	1
25–34	43	128	1.98 (1.09–3.59)	1.78 (0.08–3.78)
35–44	31	159	1.15 (0.62–2.14)	1.02 (0.68–2.45)
45–54	28	125	1.32 (0.67–2.49)	1.30 (0.69–2.58)
≥55	128	395	1.91 (1.13–3.23)	1.10 (1.69–3.54)*
Current alcohol use				
No	213	832	1	1
Yes	36	87	1.62 (1.07–2.45)	1.42 (0.91–2.09)
Family history of mental illness				
No	234	901	1	1
Yes	15	18	3.21 (1.59–6.46)	3.17 (1.54–6.96)*

*p < 0.05 and **p < 0.001. COR, crude odds ratio; AOR, adjusted odds ratio.

## Discussion

Common mental disorder is a public health problems that cause impact to the individuals, their family and communities in both developed and developing countries (26). The global burden of disease report indicate that CMD accounts around 9.8% of global burden disease (5). But the prevalence and associated factors of common mental disorders are varying among different population. This study showed that using a cut-off point >6 of SRQ-20 questionnaire; the prevalence of common mental disorder among the adult population living in the Harari regional state was 21.3% (95%CI, 18.8–23.6). The current finding was in line with the community based study conducted in Nigeria among 1,105 respondents using the 12-Item General Health Questionnaire (GHQ-12), 21.9% (27), and in Jimma, southwest Ethiopia, utilized self-reporting questionnaire-20 with cut-off point 6 out of 20 items among 1,006 individuals, 22.7% (28).

However, the result of this study was lower than another community based study conducted in the United Kingdom among 8,191 residents using the General Health Questionnaire-12 items, 24.6% (29), in Chile Santiago among 3,870 population using the Clinical Interview Schedule-Revised (CIS-R) tool, 25.5% (30), in South Africa among 209 general population according to cut-off scores of 9 and higher on the 20-item version of the Self Report Questionnaire (SRQ), 27.3% (31, 32), and in Brazil among 3,597 individuals using the Self Reporting Questionnaire (SRQ-20) with a cut-off point of seven or more, 29.9% (33). The possible reason for the discrepancy might be the data collection instruments used, this study used the Self-Reporting Questionnaire of 20-Item (SRQ-20) but the study done in South Africa used a Composite international diagnostic interview (CIDI).

On the other hand, the finding of the current study was higher than the study done in rural Kenya among 50,000 population, using the Clinical Interview Schedule-Revised (CIS-R),10.8% (6), in southern Ethiopia, among 1854 people using Composite International Diagnostic Interview (CIDI), 1.7% (34), in central Ethiopia among 10,203 urban grown-ups using the Self Reporting Questionnaire (SRQ) with a cut-off point of at least 6 out of 20 items, 11.7% (35), and in Eastern Ethiopia, among 968 residents using Self-Reported Questionnaire-20 with a cut-off point score of ≥ 6, 14.9% (14). As compared with the result of a previous study conducted in the study area, 14.9% (14) the current study result shows that the prevalence of common mental disorder is sharply increased, 21.3%; there are many possible factors that could increase the prevalence of common mental disorder in the community at the moment like the emerging novel coronavirus pandemic, ethnic war, and related socio-economic crises in Ethiopia like unemployment.

In this study, the odds of having common mental disorders were higher among female participants as compared to males. This result is supported by a study conducted in Kenya (6), South Africa (31), and a previous study done in Ethiopia (28, 35). The possible justification for this association might be due to cultural influence in which females may not discuss their problems with others as males, and it might be due to females having greater vulnerability to other psychosocial stress. The other possible justification might be related to depression in which females are 2 times more likely to have depression as compared with males (36).

Respondents who had a family history of mental illness were 3.17 times more likely to experience common mental disorders as compared to those who had no family history of mental illness. This finding was supported by a previous study carried out in Addis Ababa, Ethiopia (35). The possible reason might be due to a biological perspective or could be due to non-biological factors like sharing similar family stress and environmental factors. Additionally, as the family data indicated, if one parent has a mood disorder like depression, the child will have a risk to develop it (36).

Regarding marital status, participants who were, widowed, single, and divorced were 1.79, 2.03, and 3.06 times more likely to experience common mental disorders, respectively, as compared to participants who were married. This result was supported by the study done in Kenya (6), Britain (37), and Edmonton (38). The possible justification could be in the fact that divorced/widowed, people might be depressed due to feelings of hopelessness, worthlessness, anxiety, and doubts about solving marital problems. Another possible reason might be depression occurs most often in persons without close interpersonal relationships and in those who are divorced or single (1).

With respect to their occupational status, unemployed respondents were 1.97 times more likely to have common mental disorders as compared to governmental employed participants. This was supported by studies conducted in England (39, 40). The possible reason might be that increased job insecurity has been found to increase the chance of depressive symptoms and unemployment has been found to negatively affect self-esteem and increase feelings of distress (12). With respect to their age, the odds of having common mental disorders among participants who were age greater or equal to 55 years was 1.10 times higher as compared to respondents who were found within the age category of 15–24 years. This finding is in line with the studies in Kenya (6), and Ethiopia (35, 41). This could be due to age increasing the accumulation of stressful life events and biological changes like hormonal changes could increase and affect the emotion of individuals. Again, as age increases, more individuals tend to complain and externalize their emotional feelings (35).

## Limitations of the study

This study has some limitations. First, since the study design was cross-sectional, it cannot show the causal relationship. Second, during face-to-face interviewing the effect of the interviewer on the respondent can cause social desirability bias. Third, due to the nature of the data collection tool, recall bias can be considered a limitation. Fourth, the SRQ-20 is a screening instrument and not diagnostic of mental disorders. Fifth, the population studied were not screened for recent stressors, like loss to rule out bereavement as a confounder.

## Conclusion

The finding of this study showed that prevalence of common mental disorders was high in the community of Harari region state, Eastern Ethiopia. Being female, single, divorced, widowed, unemployed, having a family history of mental illness, and age were significantly associated with common mental disorders. Focusing on these identified associated factors, screening, early identification, and development of a program to prevent and provide appropriate home-based interventions for common mental illnesses in the community should be great attention to all stakeholders.

## Data availability statement

The raw data supporting the conclusions of this article will be made available by the authors, without undue reservation.

## Ethics statement

The studies involving human participants were reviewed and approved by Institutional Health Research Ethics Review Committee (reference number: IHRERC/043/2022) of the College of Health and Medical Sciences of Haramaya University, eastern Ethiopia. The patients/participants provided their written informed consent to participate in this study.

## Author contributions

TA contributed to conceiving the original idea, designing and conducting the study, analyzing the data, preparing, critically reviewing, and approving the manuscript for publication. AB, HK, AA, KeB, KaB, FM, DD, FW, YA, ML, DW, TB, KG, SN, AN, GD, BB, JH, AE, AG, YD, AS, AH, HA, JD, and KN contributed to designing and conducting the study, analyzing the data, and reviewing the manuscript. All authors contributed to the article and approved the submitted version.

## Conflict of interest

The authors declare that the research was conducted in the absence of any commercial or financial relationships that could be construed as a potential conflict of interest.

## Publisher’s note

All claims expressed in this article are solely those of the authors and do not necessarily represent those of their affiliated organizations, or those of the publisher, the editors and the reviewers. Any product that may be evaluated in this article, or claim that may be made by its manufacturer, is not guaranteed or endorsed by the publisher.
